# Hybrid (2D/3D) Dosimetry of Radiolabeled Gold Nanoparticles for Sentinel Lymph Node Detection in Patients with Breast Cancer

**DOI:** 10.1155/2020/2728134

**Published:** 2020-05-06

**Authors:** Gerardo Ramírez-Nava, Clara Santos-Cuevas, Guillermina Ferro-Flores, Blanca Ocampo-García, Isaac Chairez, Edgar Gómez-Argumosa, Lucero Abundiz-López, Francisco O. García-Pérez

**Affiliations:** ^1^Departamento de Materiales Radiactivos, Instituto Nacional de Investigaciones Nucleares, Ocoyoacac 52750, Estado de México, Mexico; ^2^Departamento de Bioprocesos, Instituto Politécnico Nacional, Ciudad de México 07340, Mexico; ^3^Departamento de Medicina Nuclear, Instituto Nacional de Cancerología, Ciudad de México 14000, Mexico

## Abstract

Previously, we reported the preparation and preclinical studies of ^99m^Tc-labeled gold nanoparticles-mannose (^99m^Tc-AuNP-mannose) with potential for sentinel lymph node (SLN) detection by using nuclear medicine procedures. This study aimed to evaluate the biokinetics and hybrid (2D/3D) dosimetry of ^99m^Tc-AuNP-mannose in five patients with breast cancer under a sentinel lymph node detection protocol. Anterior and posterior whole-body planar images (2D, at 0.5, 2, 6, and 24 h) and single-photon emission computed tomography (3D at 6.5 h)/computed tomography (SPECT/CT) images were acquired after ^99m^Tc-AuNP-mannose administration (37 MBq). Through a hybrid quantification method, activity in tissues of interest at the different acquisition times was determined and integrated over time to obtain the total nuclear transformations (*N*), as well as the mean residence time, in each tissue. *N* values and the OLINDA code were used for estimating the internal radiation absorbed doses. Results demonstrated that ^99m^Tc-AuNP-mannose successfully accumulates and remains up to 24 h in the sentinel lymph node without detectable migration to other lymph nodes and no side effects on patients. Negligible absorption of the radiolabeled nanoparticles into the circulatory system was observed, from which the radio-nanosystem is rapidly eliminated by kidneys. Hybrid (2D/3D) dosimetry evaluations showed equivalent doses to SLN, breast, and kidneys of 172.34, 5.32, and 0.08 mSv/37 MBq, respectively, with an effective dose of 2.05*E* − 03 mSv/MBq. The mean effective residence time in SLN was 0.92 h. This preliminary study indicates that the use of ^99m^Tc-AuNP-mannose for successful SLN detection in patients is safe, producing an effective dose at the level recommended for diagnostic studies (<10 mSv).

## 1. Introduction

In breast cancer patients, the sentinel lymph node (SLN) is defined as the first lymph node that malignant cells reach when migrating from the primary tumor [1, 2]. The histological study of the SLN for evaluation of cancer cell invasiveness is crucial in disease prognosis. For SLN detection, a blue dye or a colloid radiopharmaceutical, or both, are usually employed. Techniques for SLN detection improve the accuracy of surgical and biopsy procedures [2, 3]. However, dyes or radiopharmaceuticals currently available for clinical use are released from the SLN to other lymph nodes in a relatively short time [3].

The developments on receptor-specific/biocompatible nanoparticles (1–100 nm), useful as diagnostic, therapeutic, and drug delivery systems, have demonstrated the potential of nanotechnology in the field of biomedical imaging and medicine [4, 5]. Among others, gold nanoparticles (AuNPs) have suitable properties for many biomedical applications [6]. Recently, different systems based on AuNPs have been developed and preclinically assessed for SLN detection [5, 7, 8]. In our case, ^99m^Tc-labeled AuNP-mannose (^99m^Tc-AuNP-mannose) was prepared as a radiotracer to specifically target mannose receptors of macrophages abundantly present in the SLN [5]. Preclinical studies demonstrated that ^99m^Tc-AuNP-mannose is significantly retained in the first lymph node of Wistar rats from 1 h to at least 24 h after intradermal administration. Due to these characteristics, ^99m^Tc-AuNP-mannose could be used for SLN detection using 1-day or 2-day clinical protocols [9].

Although planar lymphoscintigraphy has been widely used for SLN detection [9, 10], the single-photon emission computed tomography (SPECT) 3D imaging, coupled with computed tomography (CT), has improved the identification of SLN in breast cancer patients [10–12]. Quantitative 3D SPECT/CT imaging is the most accurate method for evaluating dosimetry in patients; however, multiple 3D images must be acquired at numerous time points, resulting in prolonged and uncomfortable sessions for patients. Recently, hybrid planar/SPECT (2D/3D) quantification methods have been proposed to obtain biokinetic and dosimetric data of radiopharmaceuticals in a relatively short time [13, 14]. These methods employ multiple planar images to get the biodistribution models and at least one SPECT/CT image to scale the models of organs and tissues of interest to 3D by using specific imaging correction factors [13–15].

This study addresses the biokinetics and hybrid (2D/3D) dosimetry of ^99m^Tc-AuNP-mannose in five patients with breast cancer under an SLN detection protocol.

## 2. Materials and Methods

### 2.1. Preparation of ^99m^Tc-AuNP-Mannose

Technetium-99m-labeled AuNP-mannose was obtained by adding ^99m^Tc-EDDA/HYNIC-Tyr^3^-Octreotide (0.1 mL; 0.15 GBq; 0.3 *μ*moles of peptide; 1.3E14 molecules) to a sterile solution of AuNP-mannose (1.5 mL; 12 nm; 6E12 particles) prepared in a GMP-certified facility, as previously reported [16]. Radiochemical purity of >95% was corroborated by ITLC-SG/methyl-ethyl-ketone (*R*_f_ = 0.0 for ^99m^Tc-AuNP-mannose and *R*_f_ = 1.0 for ^99m^TcO_4_Na) and ultrafiltration (Amicon Ultracel, Millipore, 30,000 MW cutoff), in which ^99m^Tc-AuNP-mannose remains in the filter, whereas ^99m^Tc-EDDA/HYNIC-Tyr^3^-octreotide and ^99m^TcO_4_Na pass through the filter.

### 2.2. Clinical Studies

#### 2.2.1. Patients

After being approved by the hospital's Medical Ethics Committee, the study enrolled five female patients (Table 1), diagnosed with breast cancer (mean age ± SD, 53.60 ± 19.54 y; age range: 29–76). All patients received detailed information about the procedures and the aim of the study. Everyone agreed to participate and signed a consent form. The activity administered to each patient was 37 MBq, divided into four equal aliquots, which were injected using the intradermal periareolar technique.

#### 2.2.2. Imaging Studies

^99m^Tc-AuNP-mannose planar and SPECT/CT images were obtained to calculate the biokinetic and dosimetry parameters with a dual-head gamma camera (Symbia TruePoint SPECT/CT, Siemens), equipped with low-energy high-resolution (LEHR) collimators.

*(1) Planar Imaging*. The photopeak window was centered at 140 keV with a width of 15% (129.5–150.5 keV). To correct the photon scattering using a dual-energy window method, a lower window centered at 119 keV and a 15% width (108.5–129.5 keV), was set. The scan velocity was 12 cm/min, and the size of the matrix was 256 × 1024 pixels.

The chest and abdomen transmission factors were calculated using the ratio of the count rates  *I*_P_/*I*_WP_, obtained with a 37 MBq ^99m^Tc-filled flood source, with (*I*_P_) and without (*I*_WP_) the patient, from which the regional attenuation of the body was calculated. Anterior and posterior scintigraphy of whole-body was performed at 0.5, 2, 6, and 24 h after radiopharmaceutical administration [18, 19].

*(2) SPECT/CT*. The SPECT images were acquired using the same collimators and energy window configuration described in the previous section. Each study consisted of 120 projections covering 360°; the acquisition time of each projection was 15 seconds. The matrix size was set to 128 × 128 pixels, and the pixel size was set to 4.8 mm. The reconstruction of the nuclear images was obtained through the Flash-3D algorithm (modified form of the OSEM algorithm), considering four subsets, eight iterations, and no smoothing filter. The CT images were obtained with 130 kV and 30 mAs. The reconstruction algorithm used in these images was the filtered backprojection (FBP). The matrix size was set to 512 × 512 pixels, and pixel size was set to 1.2 mm. The thicknesses of the reconstructed slices were 1.2 and 5 mm. The CT reconstructions with slices of 1.2 mm were used to draw the regions of interest (ROIs), to obtain segmented volumes of interest (VOIs). The CT slices of 5 mm were used to get the attenuation map, in order to apply attenuation correction in the SPECT images. The SPECT/CT images of the chest and abdomen were performed 6.5 h after radiopharmaceutical administration [20, 21].

#### 2.2.3. ^99m^Tc-AuNP-Mannose Biokinetics

Both planar and SPECT images were archived in the DICOM (Digital Imaging and Communication in Medicine) format and processed with Matlab (MathWorks, 2018), ImageJ (National Institute of Health, 2016) and OsiriX MD (Pixmeo, 2019).

*(1) Planar Imaging*. The planar images were corrected by attenuation using the transmission factors *I*_P_/*I*_WP_. The scattering correction in these images was achieved with the method proposed by Koral et al. In this method, the true photopeak counts *T*_PC_  are given by the following equation [22]:

(1) $$\begin{array}{c} T_{\text{PC }}=C_{\text{PK}}-m_{\text{f}}C_{\text{ST}}, \end{array}$$

where *C*_PK_ is the total count recorded within the photopeak window, *C*_ST_ is the count within the scatter window, and *m*_f_ is a multiplying factor (*m*_f_=0.5 is commonly used for ^99m^Tc). ROIs were drawn around source organs (mammary glands, SLN, kidneys, urinary bladder, and whole-body) in each time frame. For all scans, the same set of ROIs was used, and the counts in each ROI were corrected by attenuation using the transmission factors (*I*_P_/*I*_WP_) experimentally calculated as previously mentioned, according to the conjugate-view counting method for additional scattering correction, as follows:

(2) $$\begin{array}{c} A_{\text{ROI}}=\frac{I_{\text{P}}}{I_{\text{WP}}\;}\sqrt{I_{\text{ANT}}I_{\text{POST}}}, \end{array}$$

where *A*_ROI_ is the activity in the compartment understudy, (*I*_P_/*I*_WP_) is the transmission factor experimentally calculated, and *I*_ANT_ and *I*_POST_ are the anterior and posterior counting rates, respectively. The counts were also corrected by physical decay. The activity of each organ was divided by the whole-body (WB) activity obtained from the first image acquired (100% of injected activity). The fraction of the injected activity (INA) in each source organ was calculated as follows:

(3) $$\begin{array}{c} \%\text{INA}=\frac{A_{\text{source organ }}}{A_{\text{WB at the first acquisition }}\;}\;\times 100. \end{array}$$

*(2) SPECT/CT*. The SPECT images were corrected by attenuation with the attenuation maps, which were obtained using the conversion of HU to linear attenuation coefficients. The photon scattering was corrected with a dual-energy method, which employs a single lower scatter window adjacent to the photopeak window. The scatter estimate *SE*_*PP*_ within the photopeak window is given by the following equation:

(4) $$\begin{array}{c} {\text{SE}}_{\text{PP}}=\left(\frac{W_{\text{PK}}}{2W_{{\text{ST}}_{1}}}\right)\left(P_{{\text{ST}}_{1}}\right), \end{array}$$

where *W*_PK_ and *W*_ST_1__ are the photopeak window PK widths and the scatter window ST_1_ , respectively. *P*_ST_1__ is the projection image within the scatter window ST_1_ [20].

The system sensitivity factor, *S*_SPECT_  (cps/MBq), was obtained with the following equation:

(5) $$\begin{array}{c} S_{\text{SPECT}}=\frac{{\text{CR}}_{\text{VOI}}\;\left(e\;\left(\left(T_{\text{S}}-T_{\text{CL}}\right)/\;T_{1/2}\right)\;\right)\left(T_{\text{TAT }}\ln\left(2\right)/\;T_{1/2}\;\right)}{C_{\text{Pha}}\;\left(1-e\left(-T_{\text{TAT }}\ln\left(2\right)/\;T_{1/2}\;\right)\right)}, \end{array}$$

where CR_VOI_ is the counting rate derived from the reconstructed image and the segmented VOI, *C*_Pha_ is the known activity in the phantom, *T*_S_ is the starting time of the acquisition, *T*_CL_ is the activity calibration time, *T*_1/2_ is the half-life of the radioisotope, and *T*_TAT _ is the total acquisition time of the study. To determine *S*_SPECT_, the Jaszczak Standard SPECT Phantom™ was filled with a known and uniformly distributed solution of ^99m^Tc. This experiment was carried out (*n* = 3) for activities of 37 MBq, 185 MBq, and 370 MBq (phantom concentrations of 0.005, 0.026, and 0.054 MBq/mL, respectively). *S*_SPECT_ was calculated with equation (5) and the VOIs were segmented in the reconstructed images [20, 23].

The correction factors (CF_PVE_) due to the partial volume effect (PVE) of the SPECT/CT system were calculated through a calibration method, in which five hollow spheres of different diameters were filled with equal ^99m^Tc activity concentrations (0.818, 0.409, and 0.164 MBq/mL) in a uniformly distributed background activity. This experiment was repeated for background ratios of 2 : 1, 5 : 1, and 10 : 1 (*n* = 3). The CF_PVE_ for each sphere were calculated according to the following equation [20, 24]:

(6) $$\begin{array}{c} {\text{CF}}_{\text{PVE}}=\frac{A_{\text{Activimeter }}}{A{\;}_{\text{SPECT }}}, \end{array}$$

where *A*_SPECT _ is the activity determined in the SPECT reconstructed image and *A*_Act _ is the filling activity measured with the activimeter. For the mean CF_PVE_ for each sphere, the size was calculated, and the obtained data was fitted in a function of the following equation:

(7) $$\begin{array}{c} {\text{CF}}_{\text{PVE}}\left(\text{VOI}\right)=Ae^{-a\text{VOI}}+Be^{-b\text{VOI}}+Ce^{-c\text{VOI}}, \end{array}$$

where *A*, *B*,  *C*,  *a*,  *b* , and *c* are the fitting constants and VOI is the volume of interest in the sphere under study.

The activity in the VOIs (*A*_VOI _) was calculated using the following equation:

(8) $$\begin{array}{c} A_{\text{VOI }}=\frac{R_{\text{VOI}}\;{\text{CF}}_{\text{PVE}}}{S_{\text{SPECT}}\;}, \end{array}$$

where *R*_VOI_ is the counting rate in the VOI, CF_PVE_ is the correction factor associated with the VOI, and *S*_SPECT_ is the system sensitivity factor [20, 23]. The counting rates of the SPECT images were obtained drawing ROIs in the SPECT/CT slices of the VOI under study. All the SPECT reconstructions were decay-corrected.

*(3) Hybrid Method*. Considering that SPECT/CT quantification is more accurate, correction factors between imaging modalities were calculated (equation (9)) to scale the activity obtained from planar imaging:

(9) $$\begin{array}{c} {\text{CF}}_{\text{Hyb}}=\frac{A{\;}_{\text{organ of interest in SPECT}}}{A{\;}_{\text{organ of interest in planar}}\;}, \end{array}$$

where (CF_Hyb_) are the corrections factors of the hybrid method, *A* _organ of interest in SPECT_ is the activity in the organ of interest quantified by SPECT, and *A* _organ of interest in planar_  is the activity measured in the planar images [13].

CF_Hyb_ were applied in the quantifications of the planar method (*A*(*t*)_P_) to obtain the volumetric activity quantification (*A*(*t*)_VOI_), according to the following equation:

(10) $$\begin{array}{c} A{\left(t\right)}_{\text{VOI}}={\text{CF}}_{\text{Hyb}}A{\left(t\right)}_{\text{P}}, \end{array}$$

The scaled %INAs of each organ were fitted to three-exponential models using OLINDA/EXM.

#### 2.2.4. ^99m^Tc-AuNP-Mannose Absorbed Dose Calculations

The absorbed dose to organs was evaluated according to the following equation:

(11) $$\begin{array}{c} D\left(r_{\text{T}},\;T_{\text{D}}\right)=\sum_{r_{\text{s}}}N\left(r_{\text{s}},\;T_{\text{D}}\right)\text{DF}\left(r_{\text{T}}\;⟵\;r_{\text{s}}\right), \end{array}$$

where *D*(*r*_T_,  *T*_D_) is the mean absorbed dose to a target tissue *r*_T_ from a source tissue *r*_s_,  *N*(*r*_s_,  *T*_D_) is the total number of nuclear transformations that occurred in *r*_s_ over the dose-integration period *T*_D_, and DF(*r*_T_ ⟵ *r*_s_) is the absorbed dose in *r*_T_ per nuclear transformation in *r*_s_. In this study, the equivalent absorbed dose estimates were obtained by entering the experimental  *N*(*r*_s_,  *T*_D_) values for all source organs into OLINDA/EXM [19, 25].

## 3. Results and Discussion

The SPECT detectors showed a linear response, as expected. *S*_*SPECT*_ was 572.49 cps/MBq. The CF_PVE_ fitting is given by equation (12), in which the triexponential parametric analysis yielded a correlation coefficient of *R*^2^ = 0.99:

(12) $$\begin{array}{c} {\text{CF}}_{\text{PVE}}\left(\text{VOI}\right)=1.09e^{-0.38\text{VOI}}+1.11e^{-2.68\times 10^{-3}\text{VOI}}+0.29e^{-9.61\times 10^{-5\;}\text{VOI}}. \end{array}$$

**Figure 1:**
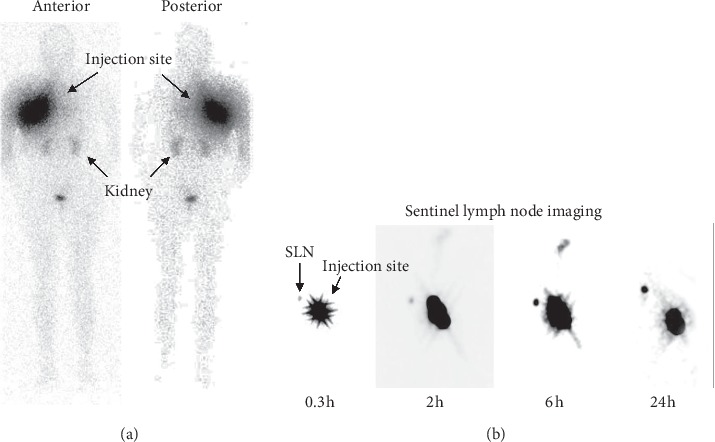
Planar images of the patient after the administration of ^99m^Tc-AuNP-mannose (37 MBq). Anterior and posterior whole-body at 2 h after radiotracer administration (a) and frontal breast view at different times (b).

**Figure 2:**
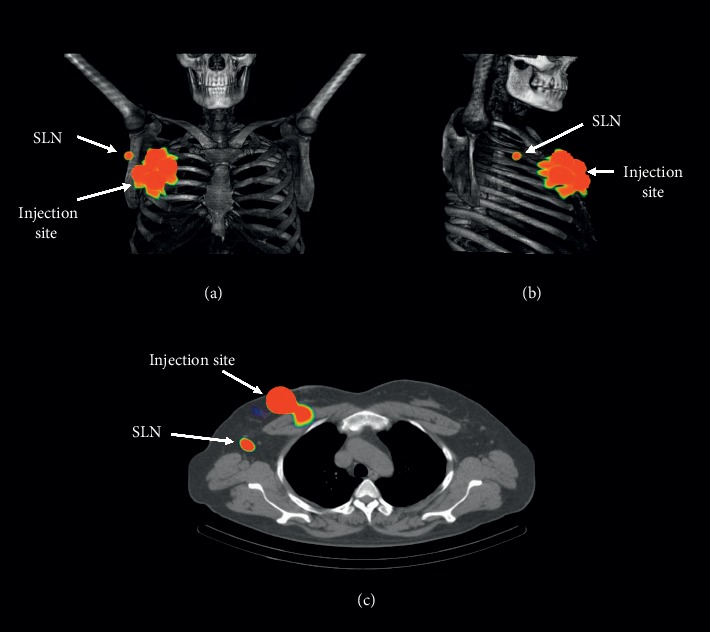
SPECT/CT imaging of the patient number 1 after 6.5 h of ^99m^Tc-AuNP-mannose (37 MBq) administration. (a) Frontal and (b) lateral views. (c) Slice of the fused SPECT/CT imaging.

Figure 1 shows the whole-body 2D images (left) and frontal 2D view of the injection site and sentinel lymph node (patient 1) acquired at different times. In this figure, only renal excretion is observed, mainly due to the radio-nanosystem functionalization with mannose [5, 16]. Figures 2(a) and 2(b) display the frontal and lateral 3D images acquired at 6.5 h after the radio-nanosystem administration.  Figure 2(c) illustrates a slice of the fused SPECT/CT imaging, where ^99m^Tc-AuNP-mannose uptake in the SLN can be easily observed.

None of the 5 patients reported side effects such as chills, muscle cramps, decreased blood pressure, bradycardia, vomiting, coughing, itching, dyspnea, bronchospasm, flushing, nausea, hives, or dizziness after the radiolabeled nanoparticles were administered. The total number of nuclear transformations that occurred in the source organs (breast, SLN, urinary bladder, and kidneys) is shown in Table 2. The equivalent radiation absorbed doses and the effective dose of ^99m^Tc-AuNP-mannose are shown in Table 3.

The effective mean residence time (∫_*t*=0_^*t*=*∞*^*A*(*t*)d*t*/*A*_0_) of the nanoparticles in the SLN was calculated to be 0.92 h, while the biological mean residence time (corrected by decay) was 6.13 h. From the latter data, the safety of unlabeled AuNP-mannose could be questioned because of the possible biological damage that could be caused by the nanoparticle itself, associated with a prolonged AuNP-tissue interaction. In this regard, it is essential to mention that the effect of nanoparticles on cells and tissues change, depending on the type of interaction at the place of contact. Several trials have demonstrated that gold nanoparticles capped with citrate (from 5 to 13 nm) caused an increase in the reactive oxygen species because AuNPs form strong Au-S bonds with intracellular glutathione and thiol-proteins [26, 27]. However, in the case of AuNPs with mannose or peptides attached to their surface, the generation of reactive oxygen species is negligible, due to the biocompatibility and steric effect induced by the biomolecules, which circumvent Au-glutathione/Au-thiol-protein reactions [27].

In this study, absorbed dose calculations were assessed using hybrid (2D/3D) dosimetry under the assumption that planar imaging (2D) methods overestimate or underestimate radiation absorbed doses due to tissue-activity overlapping or the location of small-size tissues [13, 15]. Taking 3D SPECT dosimetry as a reference, Lehnert et al. [28] demonstrated that, in ^177^Lu-based therapies, the kidney absorbed dose is overestimated by 95% when 2D planar imaging is applied and reduced to 13% when hybrid (2D/3D) dosimetry is used [28]. In another study, Koral et al. [13] observed an underestimation in the average tumor doses of small lesions in 12 patients under ^131^I-tositumomab therapy.

For comparative purposes, we also performed dosimetry calculations, eliminating 3D SPECT imaging data. In agreement with Lehnert et al. [28], a kidney radiation absorbed dose 45% higher (0.11 mSv/37 MBq) than that obtained with the 2D/3D hybrid dosimetry (0.08 mSv/37 MBq), was observed, which suggested a dose overestimation. A similar circumstance was observed for the breast and urinary bladder, where their radiation absorbed doses were overestimated by 5% (2D = 5.58 mSv/37 MBq, 2D/3D = 5.32 mSv/37 MBq) and 14% (2D = 0.12 mSv/37 MBq, 2D/3D = 0.11 mSv/37 MBq), respectively.

In contrast to the aforementioned organs, the calculated radiation absorbed dose of the SLN using 2D-dosimetry was 1.77-fold lower (97.26 mSv/37 MBq) regarding the 2D/3D hybrid dosimetry estimation (172.34 mSv/37 MBq). This SLN dose underestimation is mainly due to the limitations of planar imaging for the detection of small tissues, justifying the preference of 3D and SPECT/CT systems for its assessment [10–12].

Based on these results, it is considered that 2D/3D hybrid dosimetric calculations obtained in this research are more accurate than those assessed with the traditional 2D-conjugate-view method.

It is important to mention that the particle size of commercial ^99m^Tc-colloids used for sentinel lymph node detection is also nanometric. The effective dose of colloidal rhenium sulfide (Nanocis, particle size 8–68 nm) has been reported to be 4.7 *μ*Sv/MBq and for ^99m^Tc-DTPA-mannosyl-dextran (Lymphoseek, particle size ∼7 nm) 17.8 *μ*Sv/MBq [29, 30]. However, the effective dose of ^99m^Tc-AuNP-mannose nanoparticles (20 nm) was significantly lower (2.1 *μ*Sv/MBq) regarding Nanocis and Lymphoseek. Radiolabeled gold nanoparticles also produce lower equivalent doses in the liver (1.6 *μ*Sv/MBq) compared to Nanocis (2.8 *μ*Sv/MBq) and Lymphoseek (1.8 *μ*Sv/MBq). In kidneys, similar equivalent doses were observed between Nanocis (1.8 *μ*Sv/MBq) and ^99m^Tc-AuNP-mannose (2.0 *μ*Sv/MBq) but they were different for Lymphoseek (10 *μ*Sv/MBq) [29, 30].

## 4. Conclusions

This is the first report in which radiolabeled gold nanoparticles are applied for molecular imaging in patients. This preliminary study suggests that the use of ^99m^Tc-AuNP-mannose for SLN detection in patients is safe. The effective dose calculated by hybrid dosimetry is at the level recommended for diagnostic studies (<10 mSv).

The quantification processes based on 2D images tend to overestimate or underestimate the activity in regions and organs of interest, leading to inaccuracies at the time of the dosimetric calculations. Although these inaccuracies could be considered negligible during the assessment of diagnostic radiopharmaceuticals, in the case of therapeutic radiopharmaceuticals, treatment response of patients could be significantly affected.

## Figures and Tables

**Table 1:** Patients with breast cancer included in the SLN detection protocol with^99m^Tc-AuNP-mannose.

Patient no.	Age	Disease
1	47	Ductal carcinoma in situ
2	29	Ductal carcinoma in situ (T1a^*∗*^)
3	71	Ductal carcinoma in situ (T1b^*∗*^)
4	76	Ductal carcinoma in situ
5	45	Ductal carcinoma in situ (T1a^*∗*^)

^*∗*^According to the TNM classification of malignant tumors [17].

**Table 2:** Biokinetic model of^99m^Tc-AuNP-mannose calculated from five patients with breast cancer under an SLN protocol (37 MBq by intradermal periareolar administration in breast).

Organ	Biokinetic model *A*(*t*)_VOI_=CF_Hyb_*A*(*t*)_P_	*N*=∫_*t*=0_^*t*=*∞*^*A*(*t*)_VOI_d*t*(MBq·h/MBq) (mean ± SD)
Breasts	*A*(*t*)_VOI_=71.80*e*^−0.23*t*^+5.01*e*^−0.23*t*^+2.27*e*^−0.23*t*^ *R*^2^ = 1	3.52 *E*+0 ± 1.97*E* − 1

Kidneys	*A*(*t*)_VOI_=0.74*e*^−5.22*t*^+0.40*e*^−0.23*t*^+0.12*e*^−0.12*t*^ *R*^2^ = 1	2.85*E* − 2 ± 6.77*E* − 3

Urinary bladder	*A*(*t*)_VOI_=8.88*e*^−12.32*t*^+0.88*e*^−0.24*t*^+1 × 10^−7^*e*^−0.12*t*^ *R*^2^ = 1	4.25*E* − 2 ± 1.58*E* − 2

Sentinel lymph node	*A*(*t*)_VOI_=−66.50*e*^−0.23*t*^+54.30*e*^−0.17*t*^+12.203*e*^−0.23*t*^ *R*^2^ = 1	9.22*E* − 1 ± 2.68*E* − 1

Remainder of the body	*A*(*t*)_VOI_=21.30*e*^−0.44*t*^+2.36*e*^−0.44*t*^+0.12*e*^−0.44*t*^ *R*^2^ = 0.97	5.47*E* − 1 ± 1.74*E* − 1

**Table 3:** Average equivalent and effective doses (mSv/MBq) of^99m^Tc-AuNP-mannose, calculated from five patients with breast cancer.

Target organ	Equivalent doses (mean ± SD)	
	Hybrid 2D/3D	2D
Adrenals	1.28*E* − 03 ± 1.60*E* − 04	1.34*E* − 03 ± 1.20*E* − 04
Brain	3.46*E* − 04 ± 9.85*E* − 05	3.14*E* − 04 ± 8.08*E* − 05
Breasts	1.44*E* − 01 ± 1.00*E* − 02	1.51*E* − 01 ± 8.67*E* − 03
Gallbladder wall	1.06*E* − 03 ± 1.61*E* − 04	1.08*E* − 03 ± 1.25*E* − 04
LLI wall	5.84*E* − 04 ± 1.25*E* − 04	5.63*E* − 04 ± 1.28*E* − 04
Small intestine	5.99*E* − 04 ± 1.27*E* − 04	5.86*E* − 04 ± 1.15*E* − 04
Stomach wall	1.45*E* − 03 ± 1.77*E* − 04	1.48*E* − 03 ± 1.24*E* − 04
ULI wall	6.76*E* − 04 ± 1.37*E* − 04	6.63*E* − 04 ± 1.18*E* − 04
Heart wall	4.70*E* − 03 ± 3.94*E* − 04	4.88*E* − 03 ± 2.96*E* − 04
Kidneys	2.03*E* − 03 ± 3.31*E* − 04	3.02*E* − 03 ± 7.98*E* − 04
Liver	1.56*E* − 03 ± 1.79*E* − 04	1.60*E* − 03 ± 1.27*E* − 04
Lungs	3.61*E* − 03 ± 3.13*E* − 04	3.74*E* − 03 ± 2.31*E* − 04
Muscle	1.05*E* − 03 ± 1.30*E* − 04	1.06*E* − 03 ± 9.70*E* − 05
Ovaries	5.92*E* − 04 ± 1.28*E* − 04	5.71*E* − 04 ± 1.30*E* − 04
Pancreas	1.47*E* − 03 ± 1.84*E* − 04	1.51*E* − 03 ± 1.33*E* − 04
Red marrow	1.12*E* − 03 ± 1.38*E* − 04	1.14*E* − 03 ± 9.83*E* − 05
Osteogenic cells	2.22*E* − 03 ± 3.59*E* − 04	2.19*E* − 03 ± 2.74*E* − 04
Skin	1.38*E* − 03 ± 1.33*E* − 04	1.42*E* − 03 ± 9.54*E* − 05
Spleen	1.13*E* − 03 ± 1.47*E* − 04	1.18*E* − 03 ± 1.08*E* − 04
Thymus	4.46*E* − 03 ± 3.70*E* − 04	4.63*E* − 03 ± 2.84*E* − 04
Thyroid	8.18*E* − 04 ± 1.25*E* − 04	8.08*E* − 04 ± 9.38*E* − 05
Urinary bladder wall	2.88*E* − 03 ± 8.72*E* − 04	3.27*E* − 03 ± 1.12*E* − 03
Uterus	7.03*E* − 04 ± 1.26*E* − 04	7.04*E* − 04 ± 1.61*E* − 04
Sentinel lymph node	4.66*E* + 00 ± 6.73*E* − 01	2.63*E* + 00 ± 1.04*E* + 0
Effective dose (mSv/MBq)	2.05*E* − 03 ± 1.92*E* − 04	2.12*E* − 03 ± 1.38*E* − 04

## Data Availability

The data used to support the findings of this study is included within the article.
